# Kinetic Studies Using a Highly Sensitive Microphone Detector

**DOI:** 10.6028/jres.093.157

**Published:** 1988-12-01

**Authors:** Walter Braun, Philippe Dagaut, Barry C. Cadoff

**Affiliations:** National Institute of Standards and Technology Gaithersburg, MD 20899

**Keywords:** CO_2_ laser, energy transfer, gas phase, kinetics, measurement, optoacoustic, waveform analysis

## Abstract

A very sensitive microphone detector is used to study fast kinetic rate processes in the gas phase resulting in the generation of heat. The rate of heat evolution in turn produces a short duration pressure pulse which drives the microphone. The frequency response of the microphone is somewhat slower than required to record these pulses as they actually appear at the detector. The theory of the method used for the data reduction is presented. It is based upon the Green’s Function method which expresses the time dependent microphone signal, *X*, (*t*), as the convolution of the pressure pulse function, *f*(*t*), by the microphone’s impulse response function, *G*(*t*). A Fourier analysis of *X*(*t*) and the two relevant functions, *f*(*t*) and *G*(*t*), at a single frequency, allows direct determination of the rate constant for the kinetic process under study. The method is demonstrated by applying it to the study of vibrational energy relaxation of pentafluorobenzene in argon buffer gas and gives results in agreement with other experimental methods.

## 1. Introduction

Several direct methods have been used to study energy transfer (relaxation) processes from vibrationally excited molecules in the gas phase. These methods can be distinguished by the way the vibrationally excited molecules are produced and how they are detected. Production methods include i), electronic excitation via pulsed excimer lasers, followed by rapid internal conversion into vibrational energy or ii), direct vibrational excitation using a pulsed CO_2_ laser. Detection methods involve real-time measurement of the relaxation process through the use of a number of different energy detection probes: ultraviolet absorption, infrared absorption, infrared fluorescence, pressure wave detection (optoacoustics or interferometry), and broadening of Hg 254 nm absorption. Most of these energy detection methods are well suited to detecting relaxation in pulsed CO_2_ laser experiments when the fluence of the laser is greater than about 0.25 J/cm^2^. In experiments using excimer laser excitation the fluence is usually considerably attenuated in order to minimize photolysis by multi-photon absorption processes, resulting in a poorer signal-to-noise ratio.

Of all the detection methods employed, optoacoustic techniques (using microphones or piezoelectric crystals) are probably the most sensitive. However, microphones used in such experiments generally have a limited frequency response thereby confining measurements to systems exhibiting relatively slow energy relaxation [1]. The low pressure limit for performing such experiments is usually about 1 torr. Below this pressure the onset of dispersion (energy loss) due to diffusion and thermal conductivity results in a decreased signal. The limitation of being able to measure only slow relaxation processes has recently been circumvented by Beck, Ringwelski, and Gordon (BRG) [2]. These workers employ a small surface area piezoelectric crystal detector with a high-frequency response. Their method involves the direct monitoring of individual pressure pulses arriving at the detector as a result of the pulsed CO_2_ laser excitation of a gas. The pressure pulse is characterized by both a condensation (compression) and a rarefaction portion and their relative magnitudes is determined by the rate of energy relaxation, the size of the irradiated zone, the radiation distribution within this zone, and the speed of sound in the gas medium. The important advantage of faster response time has, however, incurred the disadvantage of lower detection sensitivity.

In this paper we explore the application of a very sensitive microphone detector to the measurement of relaxation rates that are somewhat faster than the angular frequency response of the detector. The measurements basically involve the sampling of amplitude information as well as some limited waveform information [3]. As in the BRG [2] experiments, individual pressure pulses are monitored. These pulses are not viewed as they actually appear at the detector but rather as the detector responds to them. The detector generally develops a damped ringing sine wave at a single angular frequency, *ω*_0_, termed the characteristic or natural frequency. If *ω*_0_ is much lower than the frequency of the pressure pulse then the pulse shape cannot be effectively extracted; however, amplitude and some waveform information remain so that it is still possible to measure accurate rate constants for vibrational relaxation processes faster than *ω*_0_.

Thus, the measurable range of rate constants with the present method is about the same as with the BRG [2] method. This range is limited by the approximate condition *c*_0_/(10*r*_0_)⩽*k*′*P*⩽*3c*_0_*/r*_0_, where *k*′ is the bimolecular rate constant for energy relaxation (torr s)^−1^ *P* is the bath gas pressure (torr), *c*_0_ is the speed of sound in the bath gas medium (cm/s), and *r*_0_ (cm) is the radius of the cylindrical zone irradiated by the excitation laser beam. The upper limit for measurable *k*′*P* values is approximately the reciprocal of the time it takes for a pressure pulse to traverse the diameter of the irradiated zone; the lower limit comes about because late contributions to the developing pressure wave (due to slow relaxation) ultimately interfere with earlier contributions and the resultant pressure pulse is strongly attenuated (de-phased).

## 2. Theory of the Method

### 2.1 Background

We initially follow the procedure of Rothberg et al. [4] who described the use of microphones to perform photo acoustic calorimetry. Kinetic rate constants were not obtained by these workers, but an analysis was made on how very rapid or very slow kinetic processes (compared to *ω*_0_) affect the microphone’s sensitivity. In addition, their work dealt with pulsed laser excitation of liquid phase systems while here we concentrate entirely on the gas phase.

For convenience, in the following discussion the symbol *t* is to be viewed as a retarded time. It is a measure of time that begins (*t* =0) when the pressure pulse, originating from the laser irradiation zone, arrives at the detector.

We first deal with a simple model of the detector which is taken to be that of a damped harmonic oscillator [5] and follows the differential equation,

$$u\left(d^{2}X/dt^{2}\right)+v\left(dX/dt\right)+wX=f\left(t\right),$$ (1)

where *X* is the displacement of the mechanical diaphragm from its equilibrium position, *u* is the diaphragm mass, *v* is a damping constant, and *w* is a spring constant. The functional form of a driving pressure pulse acting on the detector face as a function of time *t*, will here and in the rest of the text be given by the designation, *f*(*t*). We now employ the Green’s function method [6] to obtain the detector’s response to the driving pressure pulse. We take this to be equal to *X*(*t*) which is given by

$$X\left(t\right)=\int_{0}^{t}G\left(t,\tau \right)f\left(\tau \right)\;d\tau =G*f,$$ (2)

where

$$G\left(t,\tau \right)=A\;\sin\left(\omega_{0}\left(t-\tau \right)\right)\;\exp\left(-\beta \left(t-\tau \right)\right)$$ (3)

is the suitable Green’s function for the initial value problem given by the differential eq (1) (assuming that *X* = 0 and d*X* / dt = 0 at *t* = 0) and represents the impulse response function. Equation (3) describes an underdamped sine wave where *A* is an arbitrary amplitude constant and *β* is the damping constant. The detector response, given by eq (2) is thus the convolution of the pressure wave pulse incident on the detector, given by *f*(*τ*), by the impulse response, *G*(*t*,*τ*) and the convolution is designated by (G*f). It is understood from the limits of integration in eq (2) that *G*(*t*,*τ*) is given by eq (3) for *τ* ⩽ *t* but is zero for *τ > t.* Thus, if both *f* and *G* are known, the signal given by the detector is completely specified by eq (2). The problem, in fact, can be completely solved experimentally: if a known pressure signal *f*(*t*) is presented to the detector and its response *X*(*t*) is measured, the function G can be determined through a deconvolution process. This is in principle possible through fast Fourier transform methods (FFT). Taking the Fourier transform of eq (2) gives, by the “Convolution Theorem,”

F[X(t)]=F[G(t)]⋅F[f(t)], (4)

that is, the Fourier transform of the convolution is the product of the individual Fourier transforms. It remains to divide *F*[*X*(*t*)] by *F*[*f*(*t*)] and then, taking the inverse transform to recover *G*(*t*). Once *G*(*t*) is known, we can, in principle, recover *f*(*t*) for any unknown pressure signal. However, we should point out that the duration of pressure wave *f*(*t*) (see below) is frequently less than half of the period of the detector’s impulse response so that such a deconvolution would not be expected to cleanly recover the shape of *f*(*t*). This fact, coupled with inherent noise in the data and the use of an imperfect representation for *G*(*t*) can frequently produce unsatisfactory results. For this reason we reduce our data in a somewhat different way which we develop in the succeeding sections.

### 2.2 Development of Present Method

It would be helpful if we could separate the quantities *f* and *G* out from within the integral in eq (2). This can be achieved by taking either a single-frequency [7] Laplace or Fourier transform of eq (2). Subsequently, we will consider the latter transform as a special case of the former. Taking the Laplace transform of the convolution function as the product of the individual Laplace transforms of *G*(*t*) and *f*(*t*),

L[X(t)]=L[G(t)]⋅L[f(t)], (5)

results in

$$\int_{0}^{\infty }X\left(t\right)\exp\left(-\alpha t\right)dt=\int_{0}^{\infty }G\left(t\right)\exp\left(-\alpha t\right)dt\cdot \int_{0}^{\infty }f\left(t\right)\exp\left(-\alpha t\right)dt.$$ (6)

Here *α* is an arbitrary real and positive constant. Using the function *G*, given by eq (3), the integral containing *G* in eq (6) is given by

$$\int_{0}^{\infty }G\left(t\right)\exp\left(-\alpha t\right)dt=\omega_{0}/\left[{\left(\alpha +\beta \right)}^{2}+{\omega_{0}}^{2}\right].$$ (7)

We have thus achieved the desired separation of the two functions G and *f* in eq (2). For the special (but frequently encountered) case where the functional form of *G* (*t*) remains unchanged over a range of experimental conditions, eq (6) becomes,

$$\int_{0}^{\infty }X\left(t\right)\exp\left(-\alpha t\right)dt=\text{constant}\cdot \int_{0}^{\infty }f\left(t\right)\exp\left(-\alpha t\right)dt.$$ (8)

It then remains to reduce the actual data according to the first integral in eq (8) and calculate the second integral from known representations of *f*(*t*). These representations will, as mentioned above, be a function of the irradiation geometry and the relaxation rate constant.

The time-varying pressure, at an observation point somewhat distant from the cylindrical axis of a laser irradiated zone, as obtained from the solution of the linearized acoustic wave equation, has been given by Bailey et al. [8], with some generalization by BRG [2]. We present the results of these two papers here in a notation similar to that of BRG [2],

$$f\left(\bar{t}\right)=Q_{0}\bar{k}\int_{0}^{\infty }{\left({\bar{l}}^{2}+{\bar{k}}^{2}\right)}^{-1}\left\{-\bar{k}\exp\left[-\bar{k}\left(\bar{t}+\bar{\delta }\right)\right]\;+\bar{l}\sin\left[\bar{l}\left(\bar{t}+\bar{\delta }\right)\right]+\bar{k}\cos\left[\bar{l}\left(\bar{t}+\bar{\delta }\right)\right]\right\}\;J_{0}\left(\bar{l}\bar{r_{p}}\right)h\left(\bar{l}\right)\bar{l}d\bar{l}.$$ (9)

**Figure 1:**
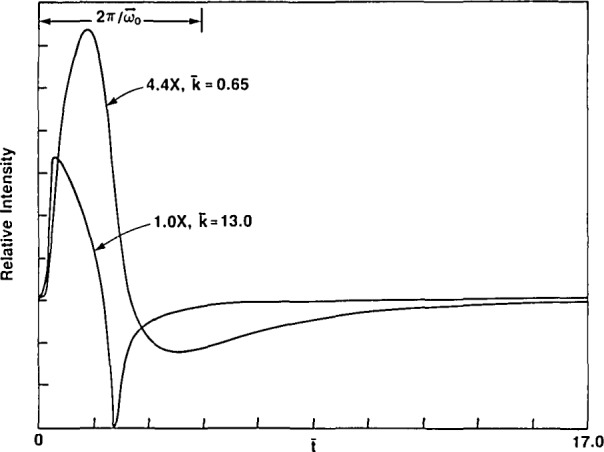
Two representative waveforms (in dimensionless units) of the pressure wave calculated from eq (9)—one for rapid relaxation, $\bar{k}=13$, and one for slower relaxation, $\bar{k}-0.65$. These two curves show approximately the full time span of the waveforms encountered under fast and relatively slow relaxation conditions. Curves are scaled by the factors indicated. The duration of one cycle of the microphone’s damped impulse response function, *G*(*t*) is shown for reference as $2\pi /{\bar{\omega }}_{0}$.

At this point we will be dealing with dimensionless quantities; the advantage of doing so will become clearer as we proceed. Also, for the sake of clarity a bar will be placed over all dimensionless quantities. In eq (9), dimensionless time is defined as $\bar{t}=tc_{0}/r_{0}$; dimensionless probe position is defined as ${\bar{r}}_{p}=r_{p}/r_{0}$; dimensionless pseudo-first-order rate constant $\bar{k}$ is given by $\bar{k}=kr_{0}/c_{0}$. The dimensional quantities consist of *r*_0_ and *c*_0_, defined earlier; *r*_p_, the probe position which is the distance of the microphone from the axis of laser beam, always ⩾*r*_0_; *k*, the pseudo-first-ordered rate constant for energy relaxation (s^−1^). This form allows us to arrive at any set of solutions regardless of the irradiation geometry and sound speed. The delay between the laser pulse and the arrival of the pressure wave at the detector is given by, $\bar{\delta }=\left(r_{p}-r_{0}\right)/r_{0}$. In eq (9), $h\left(\bar{l}\right)={\bar{J}}_{1}\left(\bar{l}\right)/\bar{l}$ characterizes a uniformly irradiated “tophat” geometry [9], *J*_0_ and *J*_1_ the zero and first order Bessel functions, respectively, and *Q*_0_ is a constant. Two examples of calculated pressure waves obtained under rapid relaxation and slow relaxation conditions are shown in figure 1.

### 2.3 Calculation of Calibration Curves

It remains to determine the integral on the right-hand side of eq (8) as a function of a variable relaxation rate constant. This integral, expressed in terms of dimensionless time, $\bar{t}$; dimensionless rate constant, $\bar{k}$, and dimensionless alpha, defined as $\bar{\alpha }=\alpha r_{0}/c_{0}$, is given by,

$$L\left[f\left(\bar{t},\bar{k}\right)\right]=\int_{0}^{\infty }f\left(\bar{t},\bar{k}\right)\exp\left(-\bar{\alpha }\bar{t}\right)d\bar{t}$$ (10)

Several curves were evaluated by numerically solving eq (10) using the expression for $f\left(\bar{t}\right)$ given by eq (9), as a function of $\bar{k}$, for several values of $\bar{\alpha }$. There is some small error involved in the numerical integration. Within this minor limitation we fit these curves (normalized to unity for the fastest relaxation rate constant) to the following empirical expression,

$$L\left[f\left(\bar{t},\bar{k}\right)\right]\propto 1-\exp\left(-\bar{a}{\bar{k}}^{0.75}\right).$$ (11)

**Figure 2:**
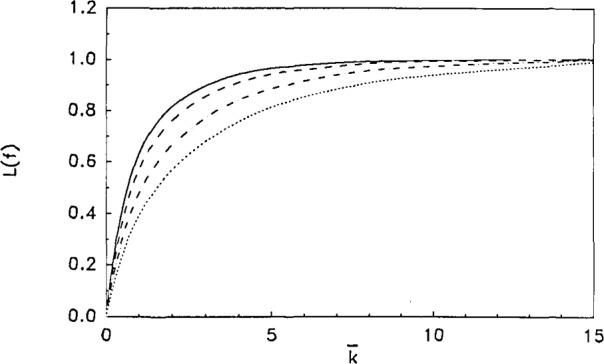
Several fits of $L\left[f\left(\bar{t}\right)\right]$ eq (10) to the functional form of eq (11) for different values of *α*. - - -, $\bar{\alpha }=0.8122,\;\bar{a}=0.85$ ___, $\bar{\alpha }=0.4060,\;\bar{a}=1.00$ - · - · -, $\bar{\alpha }=0.0812,\;\bar{a}=0.65$ · · · · ·, $\bar{\alpha }=0.0406,\;\bar{a}=0.50$

For reference, these curves are presented in figure 2. It should be noted that there may be no physical significance to the functional form given by eq (11), except that it best fits the results of the numerical integration of eq (10). Replacing the 0.75 exponent by unity results in a poorer fit. The significance of $\bar{\alpha }$ should be addressed at this point. It could first of all be viewed as a mathematical convenience allowing separation of the two terms, *f* and *G*, within the integral in eq (2). Alternatively the factor $\exp\left(-\alpha \bar{t}\right)$ in eq (10) could be viewed as a “time window” used to crudely sample the shape of the pulse (waveform) in the chosen time domain. It is ultimately expected to distinguish between different waveforms with an accuracy sufficient to return meaningful kinetic results. As we discuss below it may or may not be adequate for the problem at hand.

The results of figure 2 show that, while *α* varies by about a factor of 20, the calibration factor, $\bar{a}$, varies by only a factor of two. In order to apply this methodology it is important to note that whatever the value of $\bar{\alpha }$ used to process the actual data, the same value for $\bar{\alpha }$ must be used to calculate the appropriate calibration curve.

### 2.4 Analysis of Experimental Waveforms

We ultimately perform experiments in which a pseudo-first-order relaxation rate constant is varied linearly through a change of the bath gas pressure. The experimental signal curve is processed according to the left-hand side of eq (8),

$$L\left[X\left(\bar{t},\bar{k}\right)\right]=\int_{0}^{\infty }X\left(\bar{t},\bar{k}\right)\exp\left(-\bar{\alpha }\bar{t}\right)d\bar{t}.$$

These results must also fit the functional form (normalized to unity) of eq (11),

$$L\left[X\left(\bar{t},\bar{k}\right)\right]\propto 1-\exp\left(-AP^{0.75}\right).$$ (12)

The experimental parameter, *A*, is thus derived from the experimental data. If the functional form of $G\left(\bar{t}\right)$ remains constant, eqs (11) and (12) can simply be related according to eq (8) resulting in

$$1-\exp\left(-\bar{a}{\left({\bar{k}}^{\prime }P\right)}^{0.75}\right)=1-\exp\left(-AP^{0.75}\right),$$ (13)

where ${\bar{k}}^{\prime }=\bar{k}/P$ is the dimensionless rate constant per unit pressure (bimolecular rate constant). If the functional form of *G*(*t*) varies, eq (13) is still applicable, as discussed in section 4. However, the left-hand side of eq (12) must be replaced by *L*(*X*)/*L*(*G*). That is, the variation of *L*(*G*) must be taken into proper account. From eq (13), we arrive at

$${\bar{k}}^{\prime }={\left(A/\bar{a}\right)}^{1/0.75}.$$ (14)

Equation (14) thus involves the experimental parameter, *A*, and the appropriate calibration factor, $\bar{a}$. The dimensional rate constant per unit pressure, *k*′, is related to the dimensionless rate constant per unit pressure, ${\bar{k}}^{\prime }$, according to

$$k^{\prime }={\bar{k}}^{\prime }\left(c_{0}/r_{0}\right).$$ (15)

We have evaluated $f\left(\bar{t}\right)$ assuming that the microphone is a point detector. It actually occupies a finite width, *d*_0_, in the radial direction of the propagating pressure wave. Thus the function, $f\left(\bar{t}\right)$, should be convolved using the suitable rectangular slit function (function of ${\bar{d}}_{0}=d_{0}/c_{0}$). However, we here make the ad hoc assumption that the convolving effect of *d*_0_ on *f*(*t*) is quite analogous to the effect on *f*(*t*) produced by a simple increase in the diameter of the laser beam, *2r*_0_, which should be a good approximation if *d*_0_⩽2*r*_0_. Equation (15) is then modified to its final form,

$$k^{\prime }={\bar{k}}^{\prime }c_{0}{\left(r_{0}+d_{0}/2\right)}^{-1}={\bar{k}}^{\prime }c_{0}/r_{0\left(\text{eff}\right)},$$ (16)

where an effective *r*_0_ is calculated as indicated.

### 2.5 Fourier Transform Method

An entirely analogous treatment can be applied to the case of a Fourier transform viewed as a special case of a Laplace transform. Then for *α* = − *iω*, where $i=\sqrt{-1}$, *ω* being an arbitrary positive frequency, we refer back to eq (4), where it is understood here that we are transforming only at a single frequency, that in evaluating *F*(*X*), *F*(*G*) or *F*(*f*) the integration over time extends from zero to infinity, and that the transformed result is in the complex plane.

When *α* = *iω* eq (4) becomes

F*(X)=F*(G)F⋅F*(f). (17)

The product of eqs (4) and (17) returns a result which is a real number

F(X)⋅F*(X)=F(G)⋅F*(G)⋅F(f)⋅F*(f). (18)

Equation (18) is entirely rigorous whether *G*(*t*) remains constant or not. However, if *G*(*t*) is constant then the Fourier transform of *X*(*t*) multiplied by its complex conjugate is simply proportional to the Fourier transform of the function *f*(*t*) multiplied by its complex conjugate.

**Figure 3:**
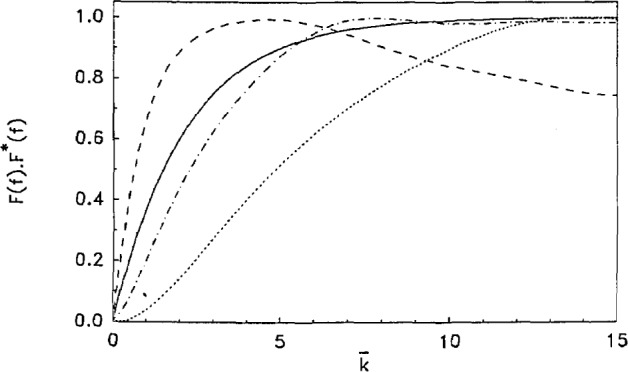
Numerical calculations *F*(*f*) · F*(*f*), eq (18), for the conditions $\bar{\omega }=1.256$ and - - -, $0.5\bar{\omega }$ - · - · -, $2.0\bar{\omega }$ · · · · ·, $4.0\bar{\omega }$ ____, $1.0\bar{\omega }$ as fit to eq (19) with $\bar{a}=0.46$.

We now develop a set of calibration curves by calculating *F*(*f*) · F*(*f*) which are the Fourier analogues of the Laplace calibrations depicted in figure 2. These are presented in figure 3 for reference. While a number of curves for variable *α* can be fit to the functional form of eq (11) for the Laplace transform case, here for the case of the Fourier transform multiplied by its complex conjugate, only a range of $\bar{\omega }$ values can be accurately fit by the following simple functional form,

$$F\left(f\right)\cdot F*\left(f\right)=1-\exp\left(-\bar{a}\bar{k}\right).$$ (19)

In all other respects the treatment given above applies to the present case, with the one exception that wherever the exponent 0.75 appears in an equation it is to be replaced by unity. Also, eq (19) should be used only for values of $\bar{\omega }$ around 1.0; for other values of $\bar{\omega }$, the other curves shown in figure 3 should be cast in suitable functional forms and counterpart experimental results analyzed in the same way.

### 2.6 Method Summary

The procedure outlined above involves taking suitable transforms, i) of the pressure wave as a function of the pseudo-first-order relaxation rate constant, ii) of *G*(*t*), if its functional form varies over the range of experimental conditions and finally, iii) of the experimental data as a function of the bath gas pressure. The processed results, when expressed in terms of the appropriate analytical functional form, allow simple extraction of the energy relaxation rate constant. Each operation involves taking a single-value transform. This accurately samples the amplitude and also samples the waveform in sufficient detail to produce accurate kinetic results.

The alternative procedure using FFT methods, as mentioned earlier, is subject to failure under the present conditions (at least with respect to recovering a precise waveform) and further requires the tedious task of making additional waveform comparisons in order to finally extract a relaxation rate constant.

## 3. Experimental Procedure

**Figure 4:**
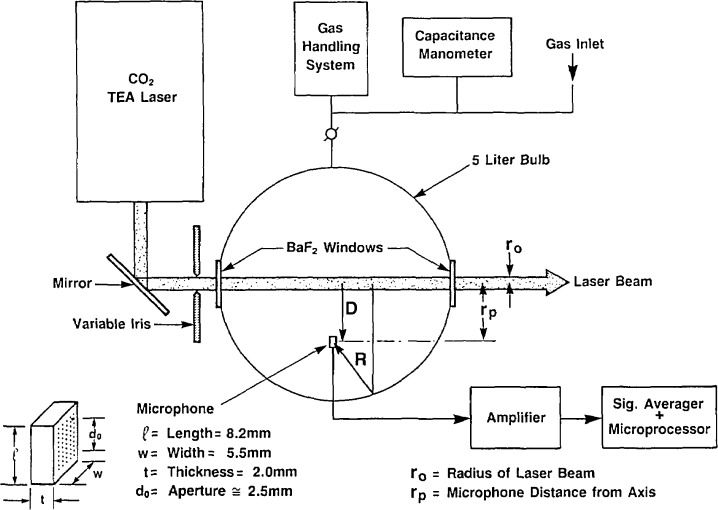
Schematic diagram of apparatus showing microphone position and approximate dimensions. The symbols D and R designate the direct (primary) and reflected pressure waves, respectively.

The essential features of the experiment are presented schematically in figure 4. A CO_2_ TEA laser delivering about 0.5 J/cm^2^ per pulse of about 200 ns duration is directed into the spherical absorption chamber through a variable iris as a quasi-parallel beam. The diameter of the laser beam is adjustable from about 2 to 10 mm. A Knowles microphone BT-1759 was used in the present work [10]. The approximate dimensions, orientation and position of the microphone are given in figure 4. The device consists of a rectangular thin-metal diaphragm placed in close proximity to an electret disk behind which is positioned a ceramic printed circuit (PC) board containing an internal amplifier. This assembly is potted within a thin metal casing, the face of which contains several rows of holes which allow the diaphragm to respond to pressure changes in the environment. An equivalent electrical circuit describing the microphone consists of a battery connected in series with a variable capacitance (the diaphragm) which is connected to the PC amplifier assembly. The assembly is biased with an external 1.35 V battery. The output of the microphone amplifier is connected with a short cable to an additional pre-amplifier which is used to reduce the output impedance of the microphone from about 3.5 kΩ to 50 Ω. The signal from this pre-amplifier is then fed to a Tektronix Model AM502 high-gain differential amplifier, the frequency response of which is set for a low-frequency 3-db cut-off of 10 KHz and a high-frequency 3-db cut-off of 1 MHz.

The output of this high gain amplifier is then connected to a Biomation Model 8100 fast digitizer which transmits the signal through an interface to the microcomputer assembly. The fast digitizer is triggered by a trigger pulse generate at the beginning of each CO_2_ laser pulse. The microphone Signal to the fast digitizer is further retarded by a pre-set time delay equal to or less than the time required for the pressure wave to travel from the laser-irradiated zone to the microphone detector. Multiple laser shots could thus be accumulated (averaged), viewed in real time, and finally stored on disks for further data reduction.

A typical set of experiments consists of i) preparing a dilute mixture (less than about one part per thousand) of an infrared absorbing gas in a non-absorbing diluent gas, in the 5-liter vessel containing the microphone assembly; ii) subjecting the mixture at some total pressure, *P*, to one or more CO_2_ laser pulses, simultaneously recording the microphone signal response and then storing the resultant waveform; iii) reducing the pressure of the mixture in stages, each followed by laser excitation and data aquisition, in order to follow the kinetics of the relaxation process. The resultant waveforms are finally processed according to the method presented in the previous section. Further details are given in section 4.

The gas handling system could be evacuated to pressures less than 1 × 10^−6^ torr. The infrared absorbing gas used in the present work was pentafluorobenzene (PFB) and the diluent gas was argon. Pressures were measured with a calibrated capacitance manometer. Gas mixtures were introduced at pressures down to about 0.5 torr and could be measured to better than 0.01 torr with the pressure measuring head.

## 4. Results

### 4.1 Microphone Response Function, *G*(*t*)

Before analyzing a typical energy relaxation experiment, we first describe several tests performed on the microphone assembly to determine its operating characteristics. These characteristics basically determine the functional form of *G*(*t*). Whatever the functional form of G (*t*), it is important that it either remain constant under the varying conditions of a set experiments, or if it varies, that its transform be independently obtained.

We first attempt to deduce an approximate functional form of *G*(*t*) for the specific microphone detector employed in these experiments and we then determine if *G*(*t*) varies under changing experimental conditions.

**Figure 5:**
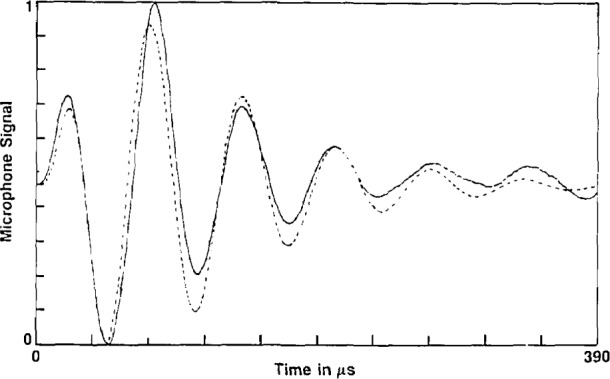
Solid (appearing) line is experimental microphone signal, 780 digitized data point, for a 4.7/1000 mixture of PFB in argon at a total pressure of 20 torr, time base extends from 0 to 390 μs. Dashed curve is a numerical calculation of *X*(*t*) using eq (2) for the function *G*(*t*)=*A* sin(*ω*_0_*t*) · [1−exp(−*γt*)] · exp(−*βt*), where *A* is arbitrary, *ω*_0_=0.097 (μs)^−1^, *γ*=0.017 (μs)^−1^, *β* = 0.014 ((μs)^−1^, and the function *f*(*t*) is taken as one cycle of a sine wave with an angular frequency of 2*ω*_0_ and arbitrary amplitude.

A computer algorithm was prepared to numerically perform the convolution given by eq (2). When we convolved *G*(*t*), given by eq (3), with a variety of simple pressure pulses, *f*(*t*), we obtained waveforms exhibiting the same characteristic feature: the first maximum was always significantly larger than the absolute value of the first minimum. The reverse effect was always observed for the experimental waveforms. Replacing eq (3) by

$$G\left(t,\tau \right)=A\;\sin\left[{\bar{\omega }}_{0}\left(t-\tau \right)\right]\left\{1-\exp\left[-\gamma \left(t-\tau \right)\right]\right\}\;\exp\left[-\beta \left(t-\tau \right)\right]$$ (20)

resulted in a much better fit as can be seen in figure 5. No attempt has been made to adjust the constants, *β* and *γ* to produce an optimum fit. In fact, it may not be possible to achieve a perfect fit to the data since the driving wave, *f*(*t*), used in the simulation consisted of a single cycle sine wave with an angular frequency, 2*ω*_0_, twice that of the detector. This idealized waveform for *f*(*t*) is only an approximation to the actual pressure waveform as shown in figure 1 (fast relaxation case). The actual detector impulse response function may also contain minor contributions at frequencies other than *ω*_0_ which are not considered here. Approximate values obtained for the parameters in eq (20) are listed in the caption of figure 5.

The following two speculative interpretations of the functional form of eq (20) are given: 1) the circuit contains an RC of about 60 μs (value of *τ* in caption of fig. 5), or 2) an initially excited microphone vibrational mode transfers energy to a second mode for which the microphone exhibits a greater response. Both explanations can account for the slowly developing signal as represented by the {1−exp[−*γ*(*t*−*τ*)} term in eq (20).

We now consider how the microphone parameters change with variation of bath gas pressure and also laser intensity.

### 4.2 Microphone Response to Laser Intensity

**Figure 6:**
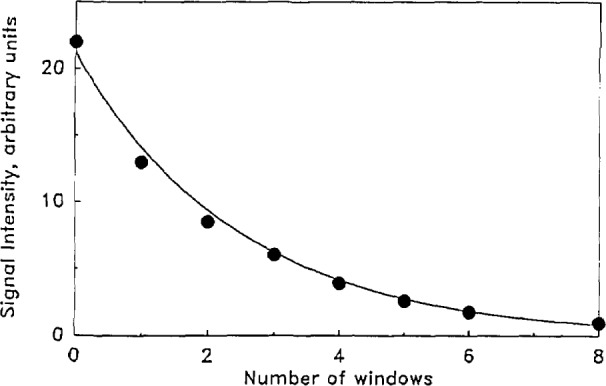
Relative intensity (of the first positive peak of the microphone signal) as a function of the number of attenuating BaF_2_ windows.

At a fixed total pressure, for a particular mixture, the laser light intensity was varied by a factor of about 100. This was effected by placing partially absorbing CaF_2_ windows in front of the laser beam incident on the spherical chamber containing the absorbing gas and microphone. Each window was expected (from previous calibrations) to attenuate the laser beam by 65 percent. Figure 6 displays results of the microphone signal, measured at the first maximum, as a function of a number of windows placed in the path of the laser. The functional form which best fits the observed relative intensity is given by

$$I=I_{0}{\left(0.663\right)}^{n},$$ (21)

where *I*_0_ is the incident laser intensity and *I* is the intensity after attenuation by *n* windows. The addition of a single window reduces the intensity by a factor of 0.663. Since the experiments are all at a constant pressure we expect that the microphone impulse function is strictly constant, and the pressure wave impinging on the microphone also has a constant waveform. Because of the latter two conditions it is necessary that the microphone produce a signal with a constant shape (waveform), regardless of magnitude. This allows us to use any part of the resultant waveform (for example, the first maximum) as a suitable measure of the laser intensity impinging on the gas mixture. In fact, a careful review of the resultant waveforms showed that the scaled waveforms could always be precisely superposed for a two orders of magnitude change in the laser intensity. The good fit of the data to the functional form of eq (21) provides equivalent information. Figure 6 further verifies that the detector is linear over the range of excitation. Our experience has shown that pressure waves too intense can cause non-linearities in either the microphone or the amplifier or both.

### 4.3 Fast Relaxation: Effect of Pressure on Microphone

**Figure 7:**
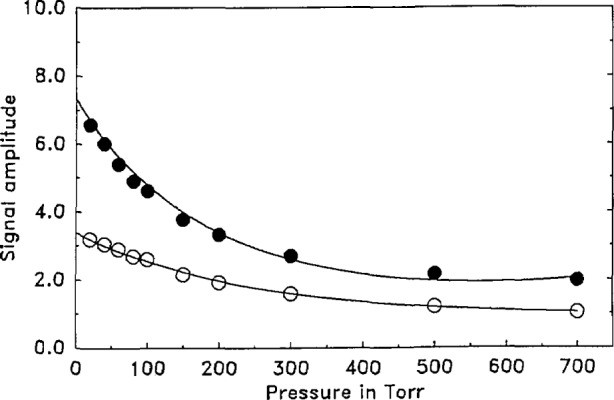
Pressure effect on microphone response: Amplitude (arbitrary units) of microphone signal sampled at two time points (*t*_0_) vs total gas pressure. First pressure consists of a 20 torr mixture of 5/1000 PFB in argon. Additional pressures obtained through consecutive additions of pure argon. Open circles (sampled at *t*_0_=26.51 μs) fit to the expression *I* = *I*_0_ exp[−1.18×10^−4^*P*−0.78×10^−7^*P*^2^) · *t*], solid circles (sampled at *t*_0_=38.34 μs) fit to the expression *I* = *I*_0_ exp[−1.22×10^−4^*P*−1.07×10^−7^*P*^2^) · *t*], with time, *t*, in μs, the pressure, *P*, in torr, *I*_0_ the amplitude extrapolated to *P*=0, and *I* the amplitude at pressure *P*.

Experiments were performed by adding additional argon to a starting mixture of about 1/1000 PFB in argon. The starting pressure was chosen high enough (20 torr) so that energy relaxation was very fast as determined from previous measurements [11]. From a pressure of 20 torr and higher, the pressure waveform, *f*(*t*), is unchanged. Assuming that the microphone impulse function, *G*(*t*), is also constant, the signal waveform must also remain unchanged. We measure the amplitude of the microphone signal at several convenient points (the first maximum, for example) and plot these in figure 7 as a function of the gas pressure. Clearly, the amplitudes are not constant showing that the functional form of *G*(*t*) does indeed vary with pressure. We suggest that this comes about because the damping constant, *β* [eq (20)] changes with pressure. On this basis the functional dependence of *G*(*t*) with pressure is obtained and given in the caption of figure 7.

It is interesting to see from figure 7 that the signal decay is not a linear function of pressure. There are, in fact, at least two decay rates, one considerably faster than the other, suggesting at least two microphone characteristic frequencies. We tentatively describe the fast decay to energy dissipation from a higher frequency mode of the microphone and the slow decay from a lower frequency mode. We cannot at this time quantitatively assess pressure changes in *γ* [eq (20)] and for present purposes assume it to be a constant.

The analysis of our experiments would be simplified if *G*(*t*) were constant with pressure. Since this is not the case, the data of figures 5 and 7 allow us to arrive at *G*(*t*) as a function of pressure through the use of eq (20) and the following derived parameters

$$\begin{array}{l} \omega_{0}=0.097{\left(\mu s\right)}^{-1},\gamma =0.017{\left(\mu s\right)}^{-1}\\ \beta =\left(0.01+1.2\times 10^{-4}P-1.0\times 10^{-7}P^{2}\right){\left(\mu s\right)}^{-1}, \end{array}$$ (20a)

where the unit of time in eq (20a) is μs and the pressure *P* is in torr.

### 4.4 Analysis of Simulated Data

Before analyzing laboratory data we describe simulated experiments in which the computer algorithm, described above, numerically calculates the convolution given by eq (2). The algorithm also numerically calculates Laplace and Fourier transforms of *X*(*t*), *G*(*t*) and *f*(*t*) performed at a single frequency, *α* or *ω.* Equations (6) and (18) were shown to be valid for a wide variety of arbitrary pressure waveforms as well as values for the parameters *α* and *ω.* That is, the transform of the “signal” was found to be equal to the product of the transform of the “pressure pulse” and the transform of *G*(*t*) as expected.

Using the above computer algorithm we were in a position to evaluate the effect of “noise” on the results of data transformed at a single frequency. Thus, we performed simulations in which random as well as systematic “noise” was added to the “signal data” to determine the effect on its transform. From these limited simulations we found (qualitatively) that introduction of systematic noise in the form of baseline shifts (which we have observed experimentally to be caused by stray electrostatic signals from the laser) as well as random noise gave results in poor agreement for the Laplace transform and in good agreement for the Fourier transform as compared with the respective noise-free results.

### 4.5 Analysis of Relaxation Data

**Figure 8:**
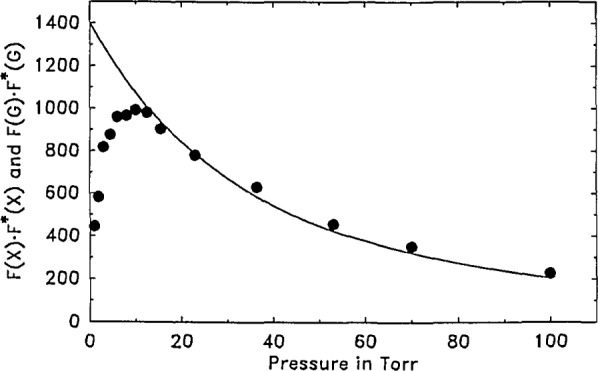
Data, *X*(*t*) transformed according to eq (18) and presented as the solid points. The similar transform of *G*(*t*) is calculated from eq (20) using the parameters listed in eq (20a). The results are displayed using the solid line.

In agreement with the above simulations we found that the experimental data transformed using the Laplace method were considerably more scattered than data using the Fourier analogue. Typical Fourier data transforms as a function of bath gas pressure are given in figure 8. By contrast, because of the poor quality of the Laplace results, we did not analyse the data by that method. However, we should point out that base line shifts in our data which we have shown to yield poor results by the Laplace method can in principle be subtracted out. This was not attempted here; however, doing so in the future may permit more effective use of the Laplace method.

We now describe the analysis of the data of figure 8 in order to obtain the rate constant for vibrational relaxation of PFB in argon. As expected, we found that the Fourier transform returns the largest numerical value when the transform is performed at *ω*_0_ and the results of figure 8 were so obtained. However, performing the transform at other frequencies is also possible.

For these experiments the laser beam dimension *r*_0_ was set at 0.42 cm. The value for *c*_0_, the sound velocity in argon, is 3.216×10^4^ cm/s, and the frequency used for the transform, *ω*, is 0.097 (μs)^−1^. These values result in $\bar{\omega }=1.256$, chosen to correspond to the simple exponential calibration curve in figure 3 $\left(\bar{a}=0.46\right)$. The experimental data, *X*(*t*) were transformed according to eq (18) and are presented as points in figure 8. Data up to about 20 torr mainly show the effect of vibrational relaxation while data at higher pressures show predominantly variation of the microphone’s impulse response function, *G*(*t*), with pressure. While pressure data as presented in figure 7, on cursory examination, suggest a moderate change of the function *G*(*t*), it is seen that the operation *F*(*G*) · F*(*G*) produces a much larger variation. However, eq (18) is entirely rigorous and is capable of handling variations in *G*(*t*) and *F*(*G*) · F*(*G*) as long as they can be quantitatively described.

There are two ways to characterize the variation of *G*(*t*) with pressure. The simplest procedure is to curve fit the transformed high pressure data in figure 8 and then assume that a short extrapolation to the low pressure regime gives the proper transform of *G*(*t*). Instead, we calculated *F*(*G*) · F*(*G*) from eqs (20) and (20a), resulting in the solid line of figure 8 [12]. This calculated curve fits the experimental points reasonably well at high pressures (where it should fit the data), and serves as a means for calculating *F*(*G*) · F*(*G*) for the low pressure points with an accuracy quite adequate for our purposes.

**Figure 9:**
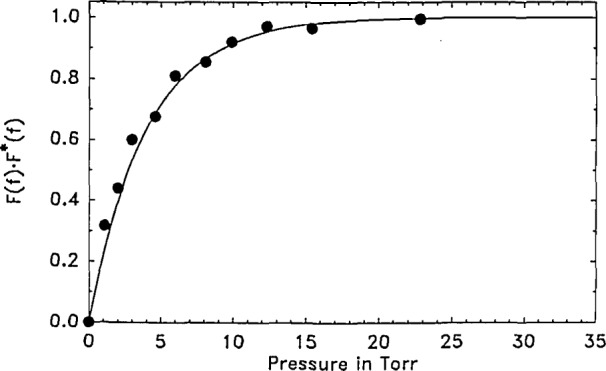
Each transformed data point at a given pressure (from fig. 8) divided by the transform of *G*(*t*) at the corresponding pressure (taken from the solid curve of fig. 8) yields, according to eq (18), the quantity of interest, *F*(*f*) · F*(*f*), and is presented as a solid point in this figure. These values, as a function of pressure, are best fit to the eq *F*(*f*) · F*(*f*)= 1−exp(−0.25· · *P*), shown as the solid line.

Finally, according to eq (18), dividing *F*(*X*) · F*(*X*) obtained at each pressure by the calculated *F*(*G*) · F*(*G*) at that pressure yields *F*(*f*) · F*(*f*), the quantity of interest. The resultant curve is presented in figure 9 where the data are fit to the functional form of eq (12) (replacing the 0.75 power by unity, for this case). The best fit yields for the parameter, *A*, the value, 0.25. According to eq (14) (again replacing 0.75 by unity) and using for $\bar{a}$ the value 0.46 results in ${\bar{k}}^{\prime }=0.54$. From eq (16), using *r*_0_=0.40 cm and *c*_0_=3.078×10^−2^ cm/μs, we derive *k* =0.042 (μs · torr)^−1^. This value is in good agreement with *k*′=0.0428±0.0092 (μs · torr)^−1^ and *k*′=0.053+0.014 (μs · torr)^−1^ obtained from a previous study employing two different methods [13].

### 4.6 Amplitude Method

**Figure 10:**
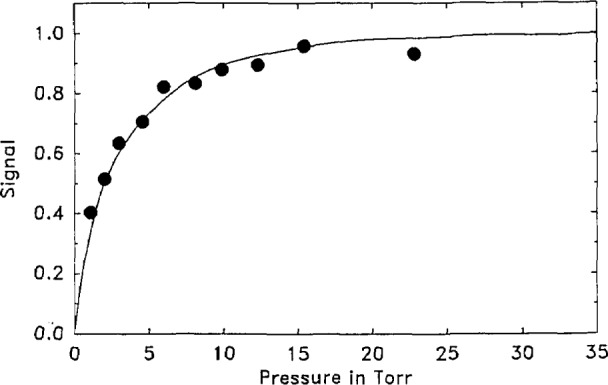
Amplitude data: Plot of the first positive peak of the microphone signal as a function of pressure (low pressure domain). Solid line is a fit of the data to the functional form, Signal = 1−exp(−*A* · *P*^0.75^), where *A* =0.40.

For comparison, the amplitude of the first maximum of the microphone signal was plotted as a function of pressure in figure 10 and the functional form [1−exp(−0.4*P*^0.75^)] was found to be a good fit to the data. The scatter in this data is somewhat greater than in the transformed data presented in figure 8. It is interesting that the amplitude plot of figure 10 is so similar to the plot of figure 9. There is nothing in the treatment presented above that would suggest that the amplitude method should give results roughly equivalent to those obtained from the transform method.

The experimental observation that amplitude data can be used in an alternative method to derive rate constants suggests that changes in the pressure pulse waveform have perhaps less influence on the overall microphone response than changes in the magnitude of the pressure pulse, at least for the present experimental conditions. In fact, we do observe that the microphone signal waveforms do not change markedly, particularly around the first maximum [14]. However, if the amplitude method is used, then the calibration constant, $\bar{a}$ in eq (14), must be derived through separate means, i.e., $\bar{a}$ cannot be obtained absolutely. That need not necessarily be a limitation if calibrations can be developed from mixtures possessing known relaxation rate constants or if the method described in the previous section provides the absolute standard. The amplitude method is simple and could be quite useful and worthy of further investigation.

## 5. Concluding Remarks

The method requires few assumptions; these can be directly examined or verified through the use of independent methods. The assumptions include: i), the function *G*(*t*) exists and ii), the signal can be represented by the simple convolution of *G*(*t*) by *f*(*t*) when terms involving the signal and its derivative at *t* =0 are equal to zero; iii), the variation of *G*(*t*) (or its transform) with pressure can be properly dealt with; and finally, iv), the representation of *f*(*t*) suitably describes the relaxation-induced pressure pulse. Assumptions i–ii have been evaluated experimentally and found to be valid; assumption iii can result in an additional source of uncertainty in the kinetic analysis if the variation of *G*(*t*) through the use of an extrapolation procedure or the use of an analytic expression is inaccurate. Other detectors may show less variation of *G*(*t*) with pressure and we plan to examine a number of these. Assumption iv can be assessed independently. For example, the functional form of *f*(*t*) has been investigated in detail by Beck and Gordon [15] and found to be an excellent representation of the experimentally derived pressure pulse. In the derivation of *f*(*t*) the additional assumption was made that first order relaxation kinetics apply. The adequacy of this assumption has been independently demonstrated for certain systems [16]. It can always be checked by simply performing kinetic measurements as a function of varying energy fluence and demonstrating the constancy of the derived “rate constant.”

In the present work we employed the Fourier transform method using a single frequency coincident with the characteristic frequency of the detector. Analysis at other frequencies are of course possible and the quality of the results obtained as a function of frequency should be addressed in more detail in future work. Additionally we plan to use the Laplace transform method in experiments that are more noise-free.

The kinetic application presented here involves the measurement of the rate of energy transfer. There are other kinetic measurements which use optoacoustic detection and involve chemical reactions. Such reactions usually generate heat and the rate of heat liberation is a measure of the rate of reaction. Recently a number of such studies have been described in the literature [4,17,18]. So far they have dealt entirely with the characterization of the extent of reaction through a procedure involving amplitude analysis which has been aptly termed photoacoustic calorimetry. The method presented here should be of value to this important new area of research since it could provide the means of performing a temporal analysis from which reaction rate constants could be derived. A microphone exhibiting very high sensitivity such as the one used here would be required. We anticipate initiating experiments of this kind.
